# Surgical and Fertility Outcomes of Reduced-Port Robotic Myomectomy: A Single-Center Experience of 401 Cases

**DOI:** 10.3390/jcm13061807

**Published:** 2024-03-21

**Authors:** Haerin Paik, Yeon Hee Hong, Yae Ji Choi, Seul Ki Kim, Jung Ryeol Lee, Chang Suk Suh

**Affiliations:** 1 Department of Obstetrics and Gynecology, Seoul National University Bundang Hospital, Seongnam 13620, Republic of Korea; 2 Department of Obstetrics and Gynecology, Seoul National University College of Medicine, Seoul 03080, Republic of Korea

**Keywords:** fertility, leiomyoma, myomectomy, pregnancy, robot surgery

## Abstract

**Background:** Reduced-port robotic myomectomy (RPRM) using Da Vinci^®^ Xi™ is a good fertility-saving surgical option, but the surgical and fertility outcomes are unknown. **Methods:** This was a retrospective cohort study evaluating the feasibility of RPRM in an academic tertiary hospital setting. A total of 401 patients who underwent RPRM by a single operator between October 2017 and October 2021 were included. For RPRM, three ports are required: a 1.5 cm umbilical incision and two 0.8 cm incisions 8 cm lateral to the umbilicus. A single-port system was applied through the umbilicus, which also functioned as a working port. Unlike conventional robotic surgery, only three robot arms were utilized for the entire surgical procedure. **Results:** Surgical and fertility outcomes were assessed through medical records review and follow-up telephone contact. The mean age of patients at the time of surgery was 39.7 ± 6.0 years. The most common indication for surgery was menorrhagia (n = 128, 31.9%). The average number of myomas removed was 4.7 ± 4.1 (1–22), and the size was 7.8 ± 2.5 cm (2.5–16.0). The mean operation time was 103.7 ± 45.6 min. Postoperative complications were found in 9.7% (n = 39) of patients; the most common complication was transfusion (7.7%, n = 31). After surgery, 70 patients tried to conceive, and 56 became pregnant naturally or by assisted reproduction (56/70, 80.0%). The mean interval time from operation to conception was 13.5 ± 10.1 months. Among 56 who conceived, 44 gave birth (62.9%), five were still ongoing (7.1%), and seven had miscarriages (10.0%). Cesarean section was performed for most cases (43/44, 97.7%). Eight patients had obstetric complications (16.3%), but no uterine rupture was reported. **Conclusions:** RPRM, which provides the benefits of conventional robotic surgery along with favorable obstetric and cosmetic results, is a feasible option for patients with symptomatic uterine myomas who wish to conceive in the future.

## 1. Introduction

Uterine myomas, also known as fibroids or leiomyomas, are the most common benign neoplasms affecting the female pelvis. In reproductive-aged females, the prevalence of myoma ranges from 20% to 75% [1,2]. Myomas originate from smooth muscle cells of the uterus and have a low mitotic index. Although benign, large myomas over 10–20 cm are occasionally seen. Myomas may cause severe menorrhagia or dysmenorrhea, urinary symptoms due to their mass effects, or gastrointestinal symptoms, affecting the quality of life. 

Myoma could affect fertility and pregnancy outcomes depending on the size and location. Intramural and submucosal myomas with intracavitary involvement in the uterus may result in lower pregnancy rates [3]. Surgical treatment may be required in specific populations to enhance pregnancy rates. For those wishing to preserve fertility, myomectomy is a feasible option. Modes of surgery could be either transabdominal (laparotomy) or laparoscopic. Compared to laparotomy, laparoscopic myomectomy has the benefits of less blood loss during the operation, shorter hospital stays, less scarring, and faster recovery [4,5]. Our group also has reported good surgical and fertility outcomes following single-port myomectomy [6]. However, no single surgical approach seems superior to others regarding the fertility rate, as available literature presents conflicting results [7]. Pitfalls of laparoscopic myomectomy include the technical difficulty of surgery and the long learning curve. 

To overcome such difficulties, robot-assisted laparoscopic surgery using da Vinci^®^ (Intuitive Surgical, Inc., Sunnyvale, CA, USA) was introduced in 1999 and gained FDA approval in 2000. It allows for three-dimensional visualization of the surgical field and flexibility by mimicking human elbow and wrist joint movements, thus enabling precise surgical works. Robot-assisted laparoscopic myomectomy (RALM) has been suggested as an effective surgical method, with comparable surgical outcomes to laparoscopic myomectomy [8]. Our group previously reported that RALM could be successfully performed in a patient with a myoma as large as 28 cm [9]. 

Conventional RALM involves four robot arms; thus, four incisions are necessary [10]. An additional incision for the working port is sometimes made, depending on the surgeon’s preference. There have been efforts to reduce port numbers in robot-assisted surgery. A concept of reduced-port robotic surgery was introduced in 2017, which involves using a single-incision laparoscopic surgery (SILS) port and an additional robotic trocar, with good surgical outcomes [11].

We developed a reduced-port robotic myomectomy (RPRM), a variation of conventional RALM, involving only three robotic arms during surgery, with one being incision-less, making it a total of three, including an umbilical incision. A commercial single-port system conventionally used for single-port surgery was used in robot surgery, along with two lateral 8 mm trocars for robotic surgery. This method may be a feasible option for women of reproductive age wishing for future fertility. This article illustrates the operative outcomes of RPRM, fertility outcomes, and possible factors affecting successful pregnancy. 

## 2. Materials and Methods

This retrospective study included 401 patients of reproductive age who underwent RPRM at Seoul National University Bundang Hospital (SNUBH) between October 2017 and October 2021. Ethical approval by the hospital’s Institutional Review Board was obtained (IRB No. 2207-769-102). We included patients of reproductive age who underwent robot-assisted myomectomy due to associated infertility or symptoms such as menorrhagia, dysmenorrhea, or discomfort due to mass effect. 

All patients underwent imaging evaluation, either via magnetic resonance imaging (MRI) or ultrasonography. A majority of patients underwent MRI for evaluation of the size and spatial relationship of myomas and to rule out the possibility of sarcomas. Robotic myomectomy was chosen for patients with submucosal myoma when the myoma was too large, so that wall defect was expected. When the size was relatively small (less than five), robotic myomectomy was performed for the following reasons: (1) concomitant intramural or subserosal myomas expediting removal were present or (2) wall defect was expected with only hysteroscopic removal, and the patient wished for future fertility.

The primary outcome of the study was fertility outcomes after RPRM, including clinical pregnancy rate and miscarriage rate. The secondary outcomes were symptomatic recurrence after surgery, postoperative complication rate, and operation-related characteristics, such as estimated blood loss (EBL) and operation time. 

Through electronic medical records, patient information, including demographic characteristics and surgical characteristics, including the average number of myomas, size, operation time, blood loss, and postoperative complication, were assessed. Symptomatic recurrence was evaluated by reviewing each patient’s medical records and ultrasonography reports. Symptoms included menorrhagia, dysmenorrhea, abdominal pain, and urinary retentions. Individual telephone contact was made to assess the pregnancy outcomes. In the case of pregnancy, additional information regarding delivery methods and postpartum complications was evaluated. 

**Figure 1:**
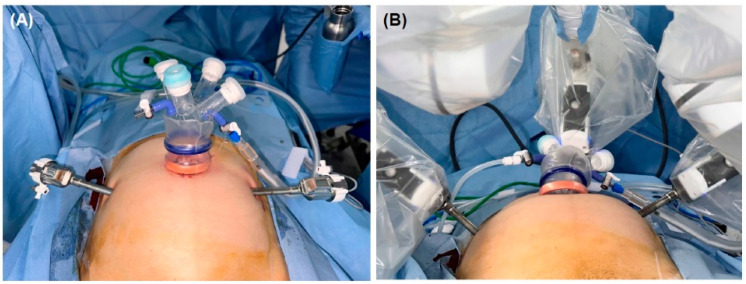
The surgical setting of the three-port robotic myomectomy (**A**) before docking and (**B**) after docking the Da Vinci^®^ Robot.

All operations were exclusively performed by a single gynecologic surgeon (JRL) with expertise in robotic surgery over 15 years. The patient was draped in a dorsal lithotomy position with arms fixed laterally to the torso. General anesthesia with intubation and total intravenous anesthesia method was applied to all patients. For the surgical process, Da Vinci^®^ Xi surgical system was used. A transumbilical incision of about 1.5–2 cm was made. The incision was made directly on the umbilicus for better cosmetic outcomes. A single-port system was applied to the transumbilical incision site. Then, under visualization with the endoscope, two 8 mm incisions about 8 cm lateral to the umbilicus were made, and robotic trocars were inserted at an appropriate depth (Figure 1). After trocar insertion and tilting the table for a trendelenberg position, a diluted vasopressin solution was injected into the myometrium to minimize blood loss. The Da Vinci^®^ patient cart was introduced to the surgical field tangentially from the left lateral aspect of the patient for robot arm docking. Three robotic arms were used for the surgery: two manipulating arms with interchangeable devices and one camera port, which was inserted transumbilically. The umbilical port was also used for manually delivering threads, removing needles, and small myomas <2 cm sized by the first assistant. 

The myoma capsule was cut with a monopolar device. When dissecting a large myoma, a diamond-shaped incision was made to facilitate the enucleation of the myoma from the capsule. Appropriate tension was maintained with Da Vinci tenaculum forceps at the left arm while dissecting with the right arm. After successful enucleation, myometrial layers were sutured using barbed sutures (v-loc™, Medtronic, Minneapolis, MN, USA) of appropriate length and thickness, depending on the situation. If the myomas were deep, the myometrial repair was performed in multiple layers, in a layer-by-layer fashion, to ensure a sound repair. An interrupted Vicryl (Vicryl™, Ethicon, Inc., Raritan, NJ, USA) suture was used to reinforce running sutures or to attain hemostasis. Specimens were removed from the abdominal cavity through the transumbilical port if myomas were less than two centimeters; if larger, myomas were removed with morcellator (Richard Wolf GmbH, Knittlingen, Germany; Karl Storz, Tuttlingen, Germany) or dissection through umbilicus within an endoscopic pouch. Power morcellation was used strictly when preoperative MRI indicated benign findings. If malignancy could not be ruled out, an intraoperative frozen biopsy was performed. An adhesion barrier was applied to all patients, and specimens were sent to the pathology department for histological evaluation. 

One day after the surgery, patients resumed their diet without checking gas passage. A postoperative complete blood count was obtained to evaluate postoperative anemia. Patients were discharged from the hospital two days after the surgery. 

We utilized Statistical Package for Social Sciences software version 26 (SPSS Inc., Chicago, IL, USA) for statistical analysis. Data were presented as mean ± SD, or median and interquartile range (IQR). The chi-square test or Fisher’s exact test was used when comparing the proportions of groups. Mann–Whitney U-test was used to compare medians of variables with nonparametric distribution. The results had statistical significance when *p*-value was less than 0.05. 

## 3. Results

The demographic characteristics of 401 patients are summarized in Table 1. The average age of enrolled patients was 39.7 ± 6.0 years, and the mean BMI was 23.0 ± 3.6 kg/m^2^. Over one-third of the population had previous abdominal surgery (39.7%). The most common indication of surgery was menorrhagia (31.9%), followed by myoma size increment (30.9%). Other causes included infertility (14.2%), chronic pelvic pain (10.2%), urinary symptoms (5.7%), and dysmenorrhea (2.2%). During surgery, an average of 4.7 ± 4.1 myomas were removed, and the mean largest diameter was 7.8 ± 2.5 cm. The most common type was intramural myomas (63.1%), followed by subserosal myomas (25.9%). We observed a small percentage of submucosal myomas (5.2%), intraligamentary myomas (2.5%), and pedunculated myomas (2.5%). The average size of submucosal myoma was 5.13 cm, and the size range was from 3 to 9.82 cm.

Operative outcomes are summarized in Table 2. The average operation time was 103.7 ± 45.6 min, and the average EBL was 149.6 ± 180.2 milliliters. The hemoglobin drop after surgery was 2.0 ± 1.0 g/dL. Patients stayed in the hospital for an average of 2.1 ± 0.6 days postoperatively. Postoperative pain scores in the numeric rating scale (NRS) were 6.2 ± 1.2 at 1 h, 3.1 ± 0.7 at 6 h, and 3.0 ± 0.7 at 24 h postoperatively. Half of the patients used continuous and interrupted methods for the suture methods (50.9%). There were no cases of conversion to laparotomy and no cases of additional trocar insertion during the operation. Eight intraoperative complications were noted, but mostly were transfusions. Postoperative complications were noted in 39 patients, the most common complication being transfusion (7.7%). According to pathologic evaluation, the mean weight of the specimen was 235.3 ± 194.4 g, and the pathology report showed leiomyoma in 96.3% and combined adenomyosis and leiomyoma in 3.7% of patients. The recurrence was assessed for 1–5 years, with a median follow-up duration of 24 months. A total of 95 patients out of 401 had medical follow-up records after at least 12 months, and 14 patients experienced symptomatic recurrence (14.7%). The median recurrence time was 13 months, and the median diameter of the recurred myoma was 3.1 cm. Reoperation was performed on seven patients (7.4%).

**Figure 2:**
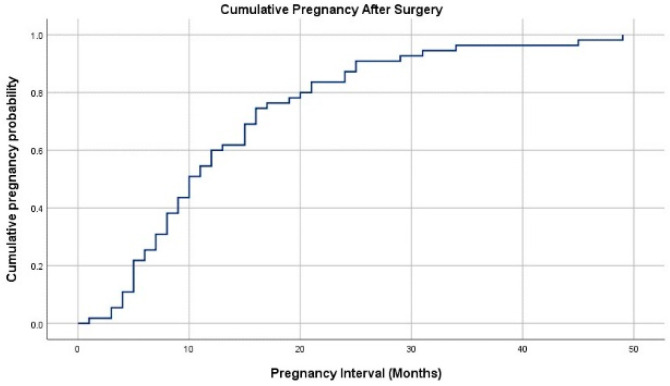
The curve for cumulative pregnancy rate.

The fertility outcomes of patients who attempted pregnancy after surgery are described in Table 3. Among 70 patients who actively tried to become pregnant, 56 (80%) became pregnant. Live birth was found in 44 patients (62.9%), ongoing pregnancy in five patients (7.1%), and miscarriage in seven patients (10.0%). The mean interval from surgery to confirmation of pregnancy was 13.5 months (Figure 2). Over half of the patients (58.9%) were pregnant with in vitro fertilization and embryo transfer (IVF-ET), and 21 patients achieved pregnancy naturally (37.5%). Twin pregnancy was noted in nine of 49 pregnancies (18.4%) that either resulted in a live birth or were ongoing. Most deliveries were at term (86.4%), and almost all deliveries were through cesarean section (97.7%), with one exception, which was a case of a patient with subserosal myoma. 

Eight patients experienced obstetric complications: six preterm labors, two premature ruptures of membranes (PPROMs), two cases of preeclampsia, and one placenta accreta. In one patient, PPROM, preterm labor, and preeclampsia all occurred; in another patient, preeclampsia and preterm labor both occurred. Three patients had postpartum complications. Two patients experienced postpartum hemorrhage, although in both cases, patients were stabilized without further intervention. Another patient experienced a remnant placenta, which was resolved by dilation and curettage. There was no uterine rupture during pregnancy. 

We compared the surgical variables of patients who became pregnant with those who did not (Table 4). The median age of patients who became pregnant was 35, and in the non-pregnant group, 38.5, although the difference was not statistically significant (*p* = 0.097). Other surgical variables of the two groups were similar. 

## 4. Discussion

In our study, the clinical pregnancy rate after reduced-port robot-assisted laparoscopic myomectomy was 80%, and the combined live birth and ongoing pregnancy rate was 70.0%, with a mean interval between surgery and pregnancy of 13.5 months. This is the highest rate among reported pregnancy rates following robot-assisted myomectomy in the available literature, which is summarized in Table 5. No uterine rupture was found among all the pregnant cases. The operative outcomes of robot-assisted laparoscopic myomectomy were also favorable, with short hospital stays (median two days), tolerable pain profiles, and low complication rates. 

The reported pregnancy rates in the current literature range from 29.1% to 77.8% with no uterine rupture [12,13,14,16,17,18,19]. Pitter et al. reported a pregnancy rate of 50.8% with a follow-up of 36 months, and the uterine rupture rate in this study was 0.9% [15]. Few studies compared fertility outcomes among different surgical methods—RALM, laparoscopic, and open myomectomy—although no differences among surgical methods have been reported so far. Flyckt et al. reported clinical pregnancy rates of 50% after RALM, 50% after laparoscopic myomectomy, and 66.7% after open myomectomy with a median of 8-year follow-up. The rates were comparable among the three groups [16]. A more recent study by Morales et al. also investigated three methods and found similar clinical pregnancy rates: 29.1% in RALM, 29.1% in laparoscopic myomectomy, and 14.3% in open myomectomy [19]. 

In our study, although patients who conceived were younger in median age compared to those who did not, it was not statistically significant. Our results indicate that the number and size of myomas removed during robotic surgery do not seem to be associated with pregnancy rates. Levobitz et al. found that age and Caucasian ethnicity are factors affecting pregnancy following myomectomy, where 11% of patients were operated on via the robotic method [20]. In studies exclusively investigating fertility after robot myomectomy, Pitter et al. reported that prior pregnancies, months since myomectomy surgery, and Caucasian race are associated with pregnancy [15]. In other studies, younger age was found to be associated with higher chances of pregnancy, and in another, a lower median number of myomas was associated with higher chances of conceiving [14,18]. Our result could be due to a small sample size with an unbalanced number in each cohort, which calls for further investigation to figure out factors associated with pregnancy outcomes in those who undergo myomectomy. 

Our reported rate of obstetric complication is similar to that observed in other studies, ranging from 0% to 17.2% [13,17,18]. Obstetric complications, including preterm labor and PPROM, could be associated with high rates of in vitro fertilization. One placenta accreta was reported in our findings, and myomectomy has been known to be a risk factor for placenta accreta [21]. Although rare, surgeons should be aware that such complications could occur. 

We observed a miscarriage rate of 10.0% (7/70), which is consistent with the rates reported in previous literature. Lonnenfors et al. observed miscarriage rates of 27.8% (5/18), including three miscarriages and two terminations [12]. Pitter et al. noted a 32.5% miscarriage rate after RALM, and Goldberg et al. reported 15.5% [15,18]. Our findings were slightly lower, even though 58.9% of our patients underwent IVF-ET procedures for pregnancy. It has been accepted that miscarriage rates after IVF are somewhat higher than normal pregnancy, although maternal age might be a risk factor [22]. In the study by Lonnenfors et al., among 15 women who became pregnant, 11 women conceived naturally [12]. Also, all miscarriages (n = 3) resulted from pregnancy after IVF. In Pitter et al.’s study, 27.7% used medications or procedures to achieve pregnancy [15]. In a study by Goldberg et al., 17.8% of patients conceived through IVF-ET and 38.5% conceived through intrauterine insemination or cycle monitoring and ovulation induction [18]. In the study by Cela et al., six out of seven women conceived naturally, and no miscarriage was reported. Huberlant et al. reported a 50% rate of pregnancy after assisted reproductive technology (IVF-ET) and 14.3% rate of miscarriage [17]. Our data, although with a higher percentage of IVF-ET pregnancies than other studies, had lower miscarriage rates. Pregnancy can be readily achieved either with IVF or spontaneously after RPRM, with low miscarriage rates. 

Our study reported higher rates of ART (58.9%) for pregnancy compared to other studies, as mentioned above. Possible reasons for such high rates could be attributed to the high volume of patients being referred to our institution from private IVF clinics for myomectomy before the IVF-ET procedure. Also, the relatively affordable cost of the IVF-ET procedure in Korea with full health insurance coverage by the government, as part of policies to cope with low fertility rates, has expedited the use of ART for pregnancy. 

Our surgical outcomes with RPRM are comparable to other studies with RALM, which incorporated four arms. The hospital stay ranged from 2 to 4.5 days, and our study had a mean postoperative hospital stay of 2.1 days. The operation time ranged from 121 to 210 min in previous literature, with the largest myoma diameter being about 6–7 cm and the average myoma count of 1 to 3.85. We reported an operation time of 103.7 min with an average myoma diameter of 7.8 cm. The average number of myomas removed in our study was 4.7, which is greater than that reported in the literature. With the RPRM method, relatively large and multiple myomas could be successfully removed without a delay in time. Blood loss was also comparable to previous studies using the RALM method. 

Regarding surgical outcomes comparing other methods of myomectomy, evidence suggests that surgical outcomes of robot or conventional laparoscopic myomectomy are more favorable than the open method. The laparoscopic method is related to less EBL, blood transfusion, and shorter hospital stays than open myomectomy. However, the operating time was longer for RALM, and the cost was higher [23]. Conventional laparoscopic myomectomy also has favorable surgical outcomes compared to laparotomy [5]. On the other hand, operative outcomes after robot and conventional laparoscopic methods seem to be similar [8]. 

Nevertheless, it should be noted that the advent of robot-assisted surgery has expanded the boundaries of laparoscopic surgery [24]. This enabled a laparoscopic approach in complex and challenging cases, in which open myomectomy would have been the only option. Surgical management of myomas through minimally invasive techniques has remained challenging to surgeons, as it involves a series of procedures, including uterine incision, enucleation, closure of myometrium, and extraction of myomas outside the pelvic cavity [25]. The introduction of robot-assisted surgery to the gynecological field made minimal invasive techniques more feasible for surgeons with enhanced dexterity during surgery. Robotic surgery translates both elbow and wrist movements into precise surgical moves, enabling appropriate tension for surgery and fine articulation for uterine incision and suture. With its ergonomic design, robot surgery helps surgeons minimize musculoskeletal fatigue with an enhanced three-dimensional view [26]. 

For the type of largest myomas in the patient population, about two-thirds had intramural myomas (63.1%). The advantage of robotic surgery could be maximized in such a setting where the dissection of deeply embedded myomas, followed by meticulous layer-by-layer repair, could be easily achieved with robotic methods with enhanced dexterity. A small portion of patients had submucosal myomas as the largest myoma; however, those patients had huge submucosal myomas with a high risk of perforation when hysteroscopically removed or multiple myomas in other locations, which are subject to removal as well. The surgery methods are individualized based on the number and location of myomas and the needs of patients. After careful consultation with each patient, we selected the best methods that fit their situation. 

This is the first report of surgical and fertility outcomes following the reduced-port setting of robot-assisted myomectomy. Good fertility outcomes could be achieved by utilizing only three robotic arms. With RPRM, a more favorable cosmesis could be expected. Arguments have been made on whether a reduced number of incisions actually benefits patients. One study reported that women do not correctly remember the number of incisions after six months; however, another study has reported that women favor fewer incisions in laparoscopic surgery [27,28]. There are more benefits than the reduced ports in RPRM. Less time is consumed for trocar insertion, docking, and closure. More importantly, surgical and fertility outcomes seem to be comparable to those reported in conventional RALM. This implies that sound myometrial repair could be performed with one arm less. We further maximized the benefits of robotic surgery by incorporating a wound retractor single-port entry system through the umbilicus and reducing one incision for better cosmetic outcomes. Conventional robotic surgery involves four to five trocar incisions, including one camera port at the umbilicus and two incisions lateral to the umbilicus [10]. Reduced-port robotic myomectomy has been attempted previously with a commercial single-port system with favorable surgical outcomes [29]. In our setting, we utilized a similar commercial single-port system with a wound retractor, which barely leaves a visible scar. 

Assistance is easier with the utilization of a commercial single-port system during surgery. Needle passages, removing small specimen fragments out of the pelvic cavity, and suction application can be readily performed through the umbilicus without consuming much time. Also, intracorporeal ultrasonography for the detection and visualization of myomas is possible through the umbilicus. If myomas were not readily identified in the operative field, intracorporeal ultrasonography was used to localize them.

Reported five-year recurrence rates after laparoscopic myomectomy range from 21.4% to 62.1% [30,31]. We reported a symptomatic recurrence rate of 14.7%, with a median of 3.1 cm. One study reported that reoperation rates after abdominal myomectomy were 12%, and our rates with robotic myomectomy were 7.4% [32]. We speculate that our recurrence data may have been over-estimated with selection bias, as those with recurrence of myomas after the operation were continuously followed up. 

For the complications, our study reported higher blood transfusion rates than those reported in other literature [33]. However, it is reasonable to attribute this to the higher number and larger size of myomas removed in our study. Also, our institutional policy is that the hemoglobin target after the operation is 10.0 mg/dL. A transfusion is performed when the preoperative hemoglobin level after the operation drops significantly compared to the preoperative status and when a patient has dizziness or shortness of breath.

Our study has strengths and limitations. We investigated fertility outcomes after reduced-port robotic myomectomy, a relatively novel method. Our data were from a single surgeon, which eliminated inter-operator variations. We also aimed to report obstetric outcomes and complications along with pregnancy rates. However, our study has several limitations. Firstly, there is an inherent fallback with the study’s retrospective nature. We aimed to overcome it with a reasonable patient cohort size and careful follow-up. Also, our study was a feasibility study, which does not provide information regarding superiority over other surgical methods. Another fallback is that our study population was relatively uniform; it was an Asian population with a relatively low BMI, so it may not be generalizable to a broader population Moreover, we could not directly compare the RPRM method with the conventional RALM method. Based on our data, we cannot prove that the surgical outcomes of RPRM are superior to RALM in terms of operative time, blood loss, and postoperative pain. Also, an analysis of factors predicting prolonged operative time and hemoglobin drop has not been made. Despite such shortcomings, our study is valuable because it is one of the few pieces of literature representing Asian women. Moreover, we could not figure out other factors that may have affected pregnancy outcomes. Further research only focused on patients undergoing robotic surgery for the sole purpose of increasing chances of fertility should be undertaken to assess pregnancy results exclusively in the infertile population. Lastly, the assessment of obstetric outcomes through telephone surveys may have been inaccurate, as recall bias may exist. However, about one-third of the patients who delivered were in our hospital; thus, acquisition of accuracy has been possible. 

In conclusion, RPRM is a feasible option for patients planning for future fertility with favorable surgical and fertility outcomes. We observed a clinical pregnancy rate of 80% among those with pregnancy intentions, the highest among currently available literature, and a combined live birth rate and ongoing pregnancy rate of 70%. Obstetric outcomes are also favorable, with no uterine rupture noted among those who delivered. Further research should focus on identifying those populations who could benefit from RPRM in terms of enhancing fertility.

## Figures and Tables

**Table 1:** Demographic characteristics of patients who underwent reduced-port robotic myomectomy.

Characteristics	Mean ± SD (Range) or n (%)
Age (years)	39.7 ± 6.0 (24–56)
BMI (kg/m^2^)	23.0 ± 3.6 (16.1–40.5)
Previous abdominal surgery	
Yes	159 (39.7%)
No	242 (60.3%)
Indication	
Menorrhagia	128 (31.9%)
Size increase	124 (30.9%)
Dysmenorrhea	9 (2.2%)
Chronic pelvic pain	41 (10.2%)
Infertility	57 (14.2%)
Urinary symptoms	23 (5.7%)
Others	19 (4.7%)
Total no. of myomas removed	4.7 ± 4.1 (1–22)
Largest diameter of myoma (cm)	7.8 ± 2.5 (2.5–16.0)
Largest myoma size (cm)	
≤5	58 (14.5%)
>5 to ≤10	284 (70.8%)
>10	59 (14.7%)
Type of largest myoma	
Submucosal	21 (5.2%)
Intramural	253 (63.1%)
Subserosal	104 (25.9%)
Intraligamentary	10 (2.5%)
Pedunculated	10 (2.5%)
Cervical	2 (0.5%)
Ectopic	1 (0.2%)

**Table 2:** Operative outcomes of reduced-port robotic myomectomy.

Operative Outcomes	Mean ± SD or n (%)
Operative time (min)	103.7 ± 45.6
Estimated blood loss (mL)	149.6 ± 180.2
Hb decrease after surgery (g/dL)	2.0 ± 1.0
Post-operative hospital stay (days)	2.1 ± 0.6
Numeric rating scale pain score	
At 1 h	6.2 ± 1.2
At 6 h	3.1 ± 0.7
At 24 h	3.0 ± 0.7
Suture methods	
Continuous	197 (49.1%)
Continuous + interrupted	204 (50.9%)
Conversion to Laparotomy	0 (0%)
Insertion of additional Port	0 (0%)
Intra-operative complications	
Yes	8 (2.0%)
No	393 (98.0%)
Post-operative complications	
Yes	39 (9.7%)
No	362 (90.3%)
Weight of specimen (g)	235.3 ± 194.4
Pathologic condition	
Leiomyoma	386 (96.3%)
Combined adenomyosis	15 (3.7%)
Recurrence Rate **	14/95 (14.7%)
Re-operation rate	7/95 (7.4%)

** Recurrence rate: symptomatic recurrence after 1–5 year of follow-up period.

**Table 3:** Fertility outcomes of patients with pregnancy intentions.

Fertility Outcomes	Mean ± SD or n (%)
No. of women of reproductive age (20–45)	327 (81.5%)
Pregnancy attempts after surgery	70
Clinical Pregnancy rate	56/70 (80.0%)
Miscarriage rate	7/70 (10.0%)
Live birth rate	44/70 (62.9%)
Ongoing pregnancy rate	5/70 (7.1%)
Interval between surgery and pregnancy (months)	13.5 ± 10.1
Method of conception	
Natural	21 (37.5%)
IUI	2 (3.6%)
IVF-ET	33 (58.9%)
Multiple gestations	
Singleton	40 (81.6%)
Twin pregnancy	9 (18.4%)
Delivery term	
Term	38 (86.4%)
Preterm	6 (13.6%)
Mode of delivery	
Vaginal delivery	1 (2.3%)
Cesarean section	43 (97.7%)
Gestational age at delivery (weeks)	37.3 ± 1.9
Birthweight (kg)	2.87 ± 0.53
Gender	
Male	29 (55.8%)
Female	23 (44.2%)
Obstetric complications *	8 (16.3%)
Postpartum complications †	3 (6.8%)
Uterine rupture rate	0 (0%)

* Obstetric complications: six preterm labors (PTLs), two premature ruptures of membranes (PPROMs), two cases of preeclampsia (PE), one placenta accreta (one patient had all PPROM, PTL, PE; another patient had PE and PTL). † Postpartum complications: two postpartum hemorrhages, one remnant placenta.

**Table 4:** Surgical variables affecting fertility.

Characteristics	Pregnant (n = 56)	Not Pregnant (n = 14)	*p*-Value
Age (years)	35 [32.25–39]	38.5 [33–41.25]	0.097 *
BMI (kg/m^2^)	22.80 [20.70–25.30]	23.43 [20.49–27.48]	0.618 *
Previous Abdominal Surgery			0.699 †
Yes	39 (69.6%)	9 (64.3%)	
No	17 (30.4%)	5 (35.7%)	
Total no. of myomas removed	4 [1–5.75]	3.5 [1–7.25]	0.89 *
Largest Diameter of myoma (cm)	6.7 [4.8–8.0]	8 [5.35–10.25]	0.311 *
Type of largest myoma			0.286 ‡
Submucosal	2 (14.3%)	2 (14.3%)	
Intramural	38 (64.3%)	9 (64.3%)	
Subserosal	16 (21.4%)	3 (21.4%)	
Operative Time (min)	127.5 [96.25–175]	135 [118.75–157.50]	0.67
Estimated blood loss (mL)	150 [100–300]	150 [100–200]	0.457
Hb decrease after surgery (g/dL)	1.9 [1.35–2.40]	2.0 [1.2–2.8]	0.994
Postop Hospital stay (days)	2 [2–2]	2 [2–2]	1
Suture methods			0.129 †
Continuous	27 (48.2%)	6 (42.9%)	
Continuous + Interrupted	29 (51.8%)	8 (57.1%)	
Complications	4 (7.1%)	0 (0%)	N/A
Weight of specimen (g)	103 [54.75–240.25]	202 [99.25–376.75]	0.13

*: Mann–Whitney U test, †: chi-square test, ‡: Fisher’s exact test. Complications: two RBC transfusions, one left sciatic nerve neuropathy, one umbilical wound dehiscence. N/A: Not available.

**Table 5:** Surgical and fertility outcomes of robot-assisted myomectomy in previous literature.

Author, Year	Study Design	Comparison Arms	Port Number	Largest Diameter	Myoma No	Myoma Weight	Hospital Stay (Day)	Operation Time (min)	Blood Loss (mL)	Follow up Duration	Clinical Pregnancy Rate	Live Birth Rate/Ongoing Pregnancy Rate	Complication Operation/Ut Rupture in Pregnancy
Lonnerfors 2011 [12]	Prospective observational study	RALM (n = 31) Deep intramural myomas	4	7 (4–11)	1 (1–5)	N/A	2 (1–3)	132 (82–213)	50 (25–200)	42 m	68%	45.5%/9.7%	22.6% (including minor ones)/0%
Cela 2013 [13]	Retrospective study	RALM (n = 48)	4	7 (1.5–9)	1 (1–7)	N/A	2 (1–3)	121 ± 46	N/A	6 m	77.8%	77.8%	N/A/0%
Tusheva 2013 [14]	Retrospective study	RALM (n = 30)	4	6.5 ± 1.7	2.0 ± 1.0	N/A	0 days (discharged 2–4 h after surg)	210.0 ± 53	100 (50–350)	N/A	75%	68.8%/0%	0%/0%
Pitter 2015 [15]	Retrospective survey	RALM (n = 426)	4	N/A	N/A	N/A	N/A	N/A	N/A	36 m	50.8%	N/A	N/A/0.9%
Flyckt 2016 [16]	Retrospective Cohort study	RALM (n = 25) LM (n = 28) Open (n = 81)	N/A	N/A	N/A	N/A	N/A	N/A	N/A	8 y	50%/50%/66.7% (NS)	50%/50%/56% (NS)	14.8% */0% vs. 0% vs. 0%
Huberlant 2019 [17]	Retrospective cohort study	RALM (n = 53)	4	6.9 ± 1.8	2 ± 1.57	173 ± 129.4	4.5 ± 0.8	N/A	260 ± 295	32 m	52.8%	41.5%	15.1%/0%
Goldberg 2022 [18]	Retrospective case series	RALM (n = 123)	4	8.9 ± 2.2	1 (1–3)	N/A	N/A	N/A	N/A	1–6 y	70.0%	63.3%, 15.5%	N/A/0% (uterine rupture)
Morales 2022 [19]	Retrospective observational study	Robotic (n = 24), Laparoscopic (n = 24), Open (n = 21) myomectomy	N/A	5.61 ± 4.63 vs. 4.22 ± 2.99 vs. 9.69 ± 7.91 (*p* = 0.004)	3.85 ± 3.06 vs. 2.56 ± 1.7 vs. 9.24 ± 8.7 (*p* = 0.000)	33.84 ± 76.70 vs. 21.6 ± 67.5 vs. 482.86 ± 1307 (*p* = 0.005)	2.0 ± 0.85 vs. 1.88 ± 0.6 vs. 2.1 ± 0.3 (*p* = 0.525)	189.85 ± 94.07 vs. 47.08 ± 98.3 vs. 42.86 ± 115.24 (*p* = 0.000)	206.54 ± 360.17 vs. 224 ± 392.14 vs. 502.86 ± 733.05 (*p* = 0.097)	N/A (Time to pregnancy: 3.87 y)	29.1% vs. 29.1% vs. 14.3%	16.7% vs. 12/5% vs. 9.5% (NS) 8.3% vs. 12.5% vs. 0%	0% vs. 0% vs. 4%/N/A
Present Study	Retrospective cohort study	RPRM (n = 401)	3	7.8 ± 2.5	4.7 ± 4.1	235.3 ± 194.4	2.1 ± 0.6	103.7 ± 45.6	149.6 ± 180.2	24 m	80.0%	62.9%/7.1%	11.2% (including minor ones)/0%

* Combined percentage of all three groups: blood transfusion (8.2%), infection (3.7%), hernia (0.7%), thromboembolism (2.2%), complication did not differ by groups. Values are expressed as mean ± SD; median (IQR) or median (range). RALM, robot-assisted laparoscopic myomectomy; N/A, not available; NS, not significant.

## Data Availability

No publicly archived datasets were analyzed nor generated during the study. The datasets used for the study are available from the corresponding author on reasonable request.
